# quincunx: an R package to query, download and wrangle PGS Catalog data

**DOI:** 10.1093/bioinformatics/btab522

**Published:** 2021-07-16

**Authors:** Ramiro Magno, Isabel Duarte, Ana-Teresa Maia

**Affiliations:** Center for Research in Health Technologies and Information Systems (CINTESIS-UAlg), University of Algarve, Faro 8005-139, Portugal; Algarve Biomedical Center (ABC), University of Algarve, Faro 8005-139, Portugal; Center for Research in Health Technologies and Information Systems (CINTESIS-UAlg), University of Algarve, Faro 8005-139, Portugal; Algarve Biomedical Center (ABC), University of Algarve, Faro 8005-139, Portugal; Center for Research in Health Technologies and Information Systems (CINTESIS-UAlg), University of Algarve, Faro 8005-139, Portugal; Algarve Biomedical Center (ABC), University of Algarve, Faro 8005-139, Portugal; Faculty of Medicine and Biomedical Sciences (FMCB), University of Algarve, Faro 8005-139, Portugal

## Abstract

**Motivation:**

The Polygenic Score (PGS) Catalog is a recently established open database of published polygenic scores that, to date, has collected, curated and made available 721 polygenic scores from over 133 publications. The PGS Catalog REST API is the only method allowing programmatic access to this resource.

**Results:**

Here, we describe *quincunx*, an R package that provides the first client interface to the PGS Catalog REST API. *quincunx* enables users to query and quickly retrieve, filter and integrate metadata associated with polygenic scores, as well as polygenic scoring files in tidy table format.

**Availability and implementation:**

*quincunx* is freely available under an MIT License, and can be accessed from https://github.com/maialab/quincunx.

**Supplementary information:**

Supplementary data are available at *Bioinformatics* online.

## 1 Introduction

For two decades, GWAS identified individual variants associated with risk for complex diseases. These associations can be combined into polygenic scores (PGS) aiming at quantifying an individual’s risk to disease, inform on prognosis and even treatment response (Lambert *et al.*, 2019). Broadly, PGS use summary statistics produced by GWAS to calculate a weighted sum of trait-associated alleles carried by each individual, in which the weights correspond to the per-allele size effects. Initially used to validate associations with disease and uncover interactions between variants, PGS have been more challenging to implement in the clinic. In 2020, over 1400 publications on PGS appeared in PubMed, raising the need for a standardized distribution of studies’ key data, assuring their wide evaluation and accurate use.

The Polygenic Score (PGS) Catalog, created in 2019, is a publicly available, manually curated database of PGS and relevant metadata, that responds to this need (Lambert *et al.*, 2020). Its current release [date 2021-02-03] includes data from 133 publications and 721 PGS associated with 194 traits. Currently, data is accessed via three ways: (i) the web graphical user interface (GUI); (ii) by downloading database dumps; and (iii) the recent PGS Catalog representational state transfer (REST) application programming interface (API), the preferred method for batch analyses.

We developed *quincunx*, the first R package (R Core Team, 2017) to programmatically access the PGS Catalog REST API. *quincunx* provides a user-friendly interface for querying the most updated Catalog data, retrieve and map it to in-memory relational databases of tidy data tables, facilitating subsequent data transformation, visualization and modelling with tidyverse packages (Wickham, 2014; Wickham and Grolemund, 2017).

## 2 Results

### 2.1 Retrieving data from the PGS Catalog REST API

The PGS Catalog REST API is an EBI service hosted at https://www.pgscatalog.org/rest/. The REST API uses hypermedia with resource responses following the OpenAPI Specification (https://swagger.io/docs/specification/about/). Response data is provided as hierarchical data in JSON format and can be paginated, i.e. split into multiple responses.

**Fig. 1.**
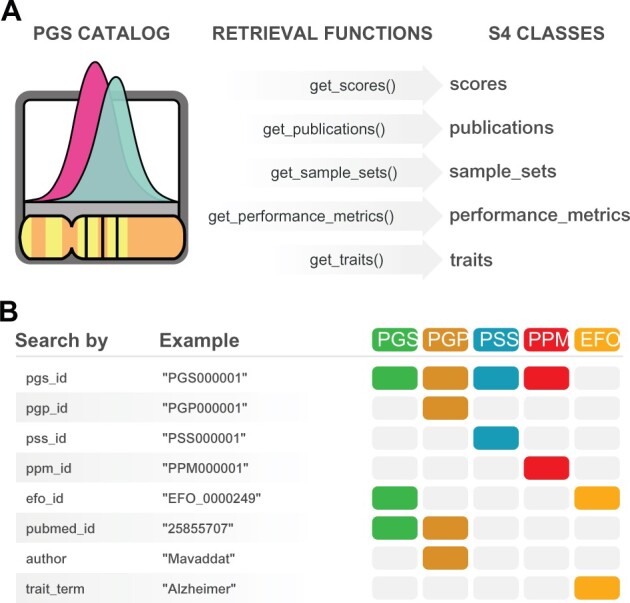
*quincunx* retrieval functions. (**A**) Functions for retrieving data from the PGS Catalog: get_scores(), get_publications(), get_sample_sets(), get_performance_metrics() and get_traits(). (**B**) *quincunx* search criteria (function parameters) to be used with retrieval functions. Coloured boxes indicate which entities can be retrieved by each search criteria

To ease the conversion from the hierarchical to the relational tabular format—the preferred format for data analysis in R (Wickham and Grolemund, 2017)—we developed a set of retrieval functions (Fig. 1A). Since the REST API data is organized around five core data entities—*Polygenic Scores*, *PGS Publications*, *PGS Sample Sets*, *PGS Performance Metrics* and *EFO traits*—we implemented five corresponding retrieval functions that encapsulate the technical aspects of resource querying and format conversion: get_scores(), get_publications(), get_sample_sets(), get_performance_metrics and get_ traits() (Fig. 1A). These functions simplify the querying of PGS Catalog entities, by providing a complete and consistent interface to the Catalog. For example, to query for *scores*, the user needs only to know the function get_scores(), whereas the REST API itself exposes three separate resource URL endpoints for *scores* with different querying parameters. Moreover, the user can choose directly the arguments of the retrieval functions from the many available search criteria exposed by the REST API (Fig. 1B). All arguments are vectorized, meaning that multiple queries are promptly available from a single function call. Results obtained from multiple queries can be combined with the logical operators OR or AND using the set_operation parameter. If set_operation is set to OR (default behaviour), results are collated while removing duplicates, if any. If set_operation is set to AND, only entities that concomitantly match all search criteria are returned. If finer control is needed on combining results, the following functions can be used: bind(), union(), intersect(), setdiff() and setequal(). These are S4 methods that work with the S4 classes created in *quincunx*. Examples of case studies (in tutorial style) can be found in Additional files 1 and 2.

### 2.2 Representation of PGS Catalog entities

All S4 classes share the same design principles that make them relational databases: (i) each slot corresponds to a table (dataframe in R); (ii) the first slot corresponds to the main table that lists observations of the respective PGS Catalog entity, e.g. *scores*; and (iii) all tables have a primary key, the identifier of the respective PGS Catalog entity: pgs_id, pgp_id, pss_id, ppm_id or efo_id. For easy consultation of the variables present in the retrieved data tables, we provide a cheatsheet (Additional file 3); and for detailed descriptions, the user can issue the help commands to open the respective help pages for each class: e.g. class? scores, class?publications, class? sample_sets.

### 2.3 Improvements and limitations

Compared to the exposed REST API, we have improved data accessibility in *quincunx* in several ways, ultimately saving human time and flattening the learning curve of the users. Firstly, we harmonized in tidy tables the nomenclature of the variables also used by the GWAS Catalog (Welter *et al.*, 2014), namely for the variables used by the R package *gwasrapidd* (Magno and Maia, 2019): an analogous R package that provides access to the GWAS Catalog REST API. Thus, permitting a frictionless wrangling of variables between the two R packages, facilitating the crosstalk between the data from the two Catalogs. We have also created the metapackage *hapiverse* (https://github.com/maialab/hapiverse) to allow simultaneous loading of the two packages. Secondly, by recognizing that in some cases the values of a variable are provided in its name and not in its value (a case of untidy data), we performed the required refactoring to make those variables explicit columns in the relational tables, thus making the data more analysis friendly. For example, the *stage* of a sample comes implicitly coded in the JSON keys samples_variants and samples_training and are mapped in *quincunx* to the variable stage, with values ‘discovery’ and ‘training’, respectively. In addition, the PGS Catalog REST API does not offer specific endpoints allowing direct mapping between the PGS entities, as this information is deeply nested in the hierarchical structure of the JSON responses. *quincunx* facilitates the retrieval of relationships between entities, by providing a set of mapping functions based on the entities’ identifiers, e.g. pgs_to_pgp, pgp_to_ppm(), ppm_to_pss(), including mapping (when applicable) from PGS scores to GWAS studies: pgs_to_study() and study_to_pgs() (see online documentation for the complete list). Finally, *quincunx* provides a set of helper functions to easily browse linked web resources, such as PubMed (open_in_pubmed()), dbSNP (open_in_dbsnp()) and the PGS Catalog Web interface itself (open_in_pgs_catalog()). More detail on the advantages of using *quincunx* can be found in the online documentation: https://maialab.org/quincunx/articles/quincunx-s-advantages.html.

## 3 Conclusion

We have developed the first R client for the PGS Catalog REST API, thus greatly facilitating the programmatic access to the database from within R. The main advantages of *quincunx* are: (i) providing a simple interface to the REST API; (ii) retrieving data in an analysis friendly format, with tidy data representations of the PGS entities, e.g, of *scores* and *performance metrics* as in-memory relational databases; (iii) allowing the automatic retrieval of polygenic scoring files from the PGS Catalog FTP server, making the data immediately available for analysis in R (not available via the REST API); and (iv) dedicating functions to export retrieved objects to Excel (.xlsx) format for data inspection and sharing outside of R. *quincunx* is a package that will greatly improve the research community’s ability for data mining within R, therefore accelerating the evaluation and subsequent application of published and manually curated polygenic scores.

## Supplementary Material

btab522_Supplementary_Data
