# Supplementary material for: quincunx: an R package to query, download and wrangle PGS Catalog data
Source: Bioinformatics. 2021 Jul 16;38(1):294–6. doi: 10.1093/bioinformatics/btab522 (PMC8696104; doi:10.1093/bioinformatics/btab522)
Supplement: btab522_Supplementary_Data [file btab522_supplementary_data.zip › additional_file_2.pdf]

Risk of Brugada syndrome patients

Introduction

In this example we walk you through an hypothetical usage case of a quincunx application with the goal of calculating the risk of developing a specific disease. For illustrative purposes, the disease chosen here is the Brugada syndrome (for details about the origin of the name, see Featuring: Josep Brugada (https://www.ecrjournal.com/articles/featuring-josep-brugada)).

Brugada syndrome is a rare, inherited cardiac disease leading to ventricular fibrillation and sudden cardiac death in structurally normal hearts. People with Brugada syndrome have an increased risk of having irregular heart rhythms beginning in the lower chambers of the heart (ventricles) (10.1038/gim.2015.35 (https://doi.org/10.1038/gim.2015.35)).

The prevalence varies among regions and ethnicities, affecting mostly males. The risk stratification and management of patients, predominantly asymptomatic, still remains challenging. Currently, despite several genes identified, SCN5A has attracted the most attention, and in approximately 30% of patients, a genetic variant may be implicated as causal factor after a comprehensive analysis (10.1016/j.jacc.2018.06.037 (https://doi.org/10.1016/j.jacc.2018.06.037)).

In the walk-through below, we show how to use quincunx to search for Brugada syndrome related polygenic scores, and how to compute the associated risk depending on the risk alleles of the associated genetic variants.

Searching for Brugada syndrome in the PGS Catalog

Before anything, let us load quincunx:

```
library(quincunx)
```

Now, we start by searching for the Brugada syndrome in the PGS Catalog. To do this we search for any traits/diseases that might include the term `"brugada"` in the trait description using the function `get_traits()`:

```
# Search in PGS Catalog for traits/diseases containing the term "brugada"
brs_traits <- get_traits(trait_term = 'brugada', exact_term = FALSE)
```

We can see that indeed there is one trait specifically associated with the Brugada syndrome, and that it has the EFO trait Orphanet_130, whose description confirms it to be a *genetic disease ventricular arrhythmia that may result in sudden death*. In addition, from the table `pgs_ids` we can retrieve the associated PGS score identifiers. To date, only one polygenic score is associated with the Brugada syndrome: PGS000737.

```
# EFO Id
brs_traits@traits$efo_id
#> [1] "Orphanet_130"

# Brugada syndrome description as defined by the Experimental Factor Ontology
brs_traits@traits$description
#> [1] "A genetically heterogeneous condition characterized by complete or incomplete
right bundle branch block accompanied by ST elevation in leads V1-V3. There is a high
incidence of ventricular arrhythmia that may result in sudden death. [NCIT: C142891]"

# PGS scores associated with Brugada syndrome
brs_traits@pgs_ids$pgs_id
#> [1] "PGS000737"
```

Getting details about the polygenic score PGS000737

From the main table, `scores`, in `brs_pgs` object, we can see the following details:

- The name given to the polygenic score PGS000737 is *PRS_BrS*
- *PRS_BrS* definition is in the PGS Catalog the same as the one in the original publication, because `matches_publication` is `TRUE`
- The original authors' reported trait was *Brugada syndrome*
- And finally, the number of variants comprising *PRS_BrS* is three.

```
brs_pgs <- get_scores(pgs_id = 'PGS000737')

# PGS code name
brs_pgs@scores$pgs_name
#> [1] "PRS_BrS"

# Does the PGS score matches the one in the source publication?
brs_pgs@scores$matches_publication
#> [1] TRUE

# Authors' own description of the trait
brs_pgs@scores$reported_trait
#> [1] "Brugada syndrome"

# Number of variants included in the polygenic risk score
brs_pgs@scores$n_variants
#> [1] 3
```

Original publication that developed PGS000737

From the table `publications` , in `brs_pgs` object, we can see, *inter alia*, the following details about the publication behind the PGS PGS000737:

- The Catalog identifier assigned to this publication is PGP000144
- The publication PubMed id is 31504448`
- It has been published in 2019-10-01
- The publication title is: Predicting cardiac electrical response to sodium-channel blockade and Brugada syndrome using polygenic risk scores.

```
# PGP id
brs_pgs@publications$pgp_id
#> [1] "PGP000144"

# PubMed id
brs_pgs@publications$pubmed_id
#> [1] "31504448"

# Publication date
brs_pgs@publications$publication_date
#> [1] "2019-10-01"

# Journal
brs_pgs@publications$publication
#> [1] "Eur Heart J"

# Title
brs_pgs@publications$title
#> [1] "Predicting cardiac electrical response to sodium-channel blockade and Brugada
syndrome using polygenic risk scores."
```

To view this publication online: `open_in_pubmed(brs_pgs@publications$pubmed_id)` .

Sample ancestry

To get a feeling for the applicability of this polygenic score, it is important to know the genetic diversity of the participants involved in the development of the risk score. We can get details on the ancestry composition of the samples used to develop PRS_BrS by looking into the `samples` table. In this table we can see that one sample (`sample_id = 1`) of 1427 individuals was used at the gwas stage. The ancestry category associated with these individuals is European. You can use quincunx's internal dataset on possible ancestry categories: `ancestry_categories` .

From this data, one may assume that the applicability of this risk score should probably be restricted to people from European ancestry.

```
# Stage
brs_pgs@samples$stage
#> [1] "gwas"

# Sample size
brs_pgs@samples$sample_size
#> [1] 1427

# Ancestry
brs_pgs@samples$ancestry_category
#> [1] "European"

# Definition of European ancestry
ancestry_categories[ancestry_categories$ancestry_category == 'European', 'definition'
, drop = TRUE]
#> [1] "Includes individuals who either self-report or have been described by authors
as European, Caucasian, white, or one of the sub-populations from this region (e.g.,
 Dutch). This category also includes individuals who genetically cluster with referen
ce populations from this region, for example, 1000 Genomes and/or HapMap CEU, FIN, GB
R, IBS, and TSI populations"

# Cohort
brs_pgs@cohorts
#> # A tibble: 1 x 4
#>   pgs_id   sample_id cohort_symbol cohort_name
#>   <chr>        <int> <chr>         <chr>
#> 1 PGS0007…         1 DESIR         Epidemiological Study on the Insulin Resista…
```

The genetic composition of PRS_BrS

To get the polygenic risk score *PRS_BrS* (i.e., PGS000737), we start by downloading it using the function `read_scoring_file`:

```
PRS_BrS <- read_scoring_file('PGS000737')
PRS_BrS
#> $PGS000737
#> $PGS000737$metadata
#> # A tibble: 1 x 7
#>   pgs_id  reported_trait  original_genome… number_of_varia… pgp_id citation
#>   <chr>   <chr>           <chr>                       <int> <chr>  <chr>
#> 1 PGS000… Brugada syndro… GRCh37                          3 PGP00… Tadros R et …
#> # … with 1 more variable: license <chr>
#>
#> $PGS000737$data
#> # A tibble: 3 x 8
#>   rsID       chr_name chr_position effect_allele reference_allele effect_weight
#>   <chr>      <chr>           <int> <chr>         <chr>                    <dbl>
#> 1 rs11708996 3            38633923 C             G                         0.55
#> 2 rs10428132 3            38777554 G             T                        -0.94
#> 3 rs9388451  6           126090377 C             T                         0.46
#> # … with 2 more variables: weight_type <chr>, locus_name <chr>
```

This PGS is comprised of only three variants: rs11708996, rs10428132, and rs9388451. These variants are located near loci well-know to be associated with the Brugada syndrome: SCN5A, SCN10A, NCOA7, and HEY2. From the `effect_weight` column, we can see that the effect allele (`"C"` in both cases) of variants rs11708996 and rs9388451 increases the risk score ($\beta = 0.55$ and $\beta = 0.46$, respectively), whereas the `"G"` allele of rs10428132 has a protective effect relative to the `"T"` allele ($\beta = -0.94$).

Now, to illustrate the calculation of *PRS_BrS* using appropriate patient-level data, we will generate all possible genotypes for these three variants, and calculate their respective risk scores. (We are not using real patient data here since such human datasets are not open). Each biallelic variant has three possible genotypes: A/A, A/a or a/a. Given that the score is also comprised of three variants, the total number of possible genotypes is $3 \times 3 \times 3 = 27$.

The function `genotypes` defined below generates the three genotypes for the given variant alleles:

```
genotypes <- function(allele1, allele2) {
  alleles <- c(allele1, allele2)
  m <- tidyr::expand_grid(allele1 = alleles, allele2 = alleles)[-3,]
  paste(m[, 1, drop = TRUE], m[, 2, drop = TRUE], sep = '/')
}
```

For example, rs11708996 genotypes are:

```
genotypes('G', 'C')
#> [1] "G/G" "G/C" "C/C"
```

The set of all 27 genotypes can be generated as follows:

```
BrS_genotypes <- tidyr::expand_grid(
  rs11708996 = genotypes('G', 'C'),
  rs10428132 = genotypes('T', 'G'),
  rs9388451 = genotypes('T', 'C')
) %>%
  tibble::rowid_to_column('patient')

print(BrS_genotypes, n = Inf)
#> # A tibble: 27 x 4
#>    patient rs11708996 rs10428132 rs9388451
#>      <int> <chr>      <chr>      <chr>
#>  1       1 G/G        T/T        T/T
#>  2       2 G/G        T/T        T/C
#>  3       3 G/G        T/T        C/C
#>  4       4 G/G        T/G        T/T
#>  5       5 G/G        T/G        T/C
#>  6       6 G/G        T/G        C/C
#>  7       7 G/G        G/G        T/T
#>  8       8 G/G        G/G        T/C
#>  9       9 G/G        G/G        C/C
#> 10      10 G/C        T/T        T/T
#> 11      11 G/C        T/T        T/C
#> 12      12 G/C        T/T        C/C
#> 13      13 G/C        T/G        T/T
#> 14      14 G/C        T/G        T/C
#> 15      15 G/C        T/G        C/C
#> 16      16 G/C        G/G        T/T
#> 17      17 G/C        G/G        T/C
#> 18      18 G/C        G/G        C/C
#> 19      19 C/C        T/T        T/T
#> 20      20 C/C        T/T        T/C
#> 21      21 C/C        T/T        C/C
#> 22      22 C/C        T/G        T/T
#> 23      23 C/C        T/G        T/C
#> 24      24 C/C        T/G        C/C
#> 25      25 C/C        G/G        T/T
#> 26      26 C/C        G/G        T/C
#> 27      27 C/C        G/G        C/C
```

In `BrS_genotypes` we have now a table of all possible genotypes comprising the three variants of *PRS_BrS*. We have prepended a column with a dummy patient identifier (`patient`) to refer to each one of the 27 possible genotypes in an unambiguous way.

PRS_BrS calculation

If we assume an additive genetic architecture, with independence of risk variants, then the calculation of PRS_BrS is straightforward: simply multiply the number of effect alleles in the genotype by the `effect_weight` , and add up everything:

$$PRS_{BrS} = \beta_{\text{rs11708996}} \times x_{rs11708996} + \beta_{\text{rs10428132}} \times x_{rs10428132} + \beta_{\text{rs9388451}} \times x_{rs9388451}$$

where, e.g., $\beta_{\text{rs11708996}} = 0.55$, and $x_{rs11708996}$ is either 0, for genotype “G/G”, 1, for genotype “G/C” or 2, for genotype “C/C”.

As a side note, although simplistic, the additive model reflects our best estimate of genetic architecture of common complex disorders, where little evidence of interaction between genetic variants is detected. And although this type of model ignores any gene-gene or gene-environment interactions (10.1002/gepi.21989 (https://doi.org/10.1002/gepi.21989)), it seems, nevertheless, that the largest meta-analysis of heritability from twin studies supports a simple additive model in most of the traits examined (10.1038/ng.3285 (https://doi.org/10.1038/ng.3285)).

Next we calculate PRS_BrS for the genotypes `BrS_genotypes` :

```
prs <- Vectorize(function(scoring, variant_id, genotype) {
  alleles <- unlist(strsplit(genotype, '/'))
  eff <-
    scoring[scoring$rsID == variant_id, 'effect_allele', drop = TRUE]
  weight <-
    scoring[scoring$rsID == variant_id, 'effect_weight', drop = TRUE]

  (alleles[1] == eff) * weight + (alleles[2] == eff) * weight
}, vectorize.args = 'genotype')
```

And, *voilà*, here are the risk scores for all possible genotypes:

```
s <- PRS_BrS$PGS000737$data
scores <- BrS_genotypes %>%
  dplyr::mutate(
    score = prs(s, 'rs11708996', rs11708996) +
      prs(s, 'rs10428132', rs10428132) +
      prs(s, 'rs9388451', rs9388451)
  )

print(scores, n = Inf)
#> # A tibble: 27 x 5
#>    patient rs11708996 rs10428132 rs9388451   score
#>      <int> <chr>      <chr>      <chr>       <dbl>
#>  1       1 G/G        T/T        T/T        0
#>  2       2 G/G        T/T        T/C        0.46
#>  3       3 G/G        T/T        C/C        0.92
#>  4       4 G/G        T/G        T/T       -0.94
#>  5       5 G/G        T/G        T/C       -0.48
#>  6       6 G/G        T/G        C/C       -0.0200
#>  7       7 G/G        G/G        T/T       -1.88
#>  8       8 G/G        G/G        T/C       -1.42
#>  9       9 G/G        G/G        C/C       -0.96
#> 10      10 G/C        T/T        T/T        0.55
#> 11      11 G/C        T/T        T/C        1.01
#> 12      12 G/C        T/T        C/C        1.47
#> 13      13 G/C        T/G        T/T       -0.39
#> 14      14 G/C        T/G        T/C        0.0700
#> 15      15 G/C        T/G        C/C        0.53
#> 16      16 G/C        G/G        T/T       -1.33
#> 17      17 G/C        G/G        T/C       -0.87
#> 18      18 G/C        G/G        C/C       -0.410
#> 19      19 C/C        T/T        T/T        1.1
#> 20      20 C/C        T/T        T/C        1.56
#> 21      21 C/C        T/T        C/C        2.02
#> 22      22 C/C        T/G        T/T        0.160
#> 23      23 C/C        T/G        T/C        0.62
#> 24      24 C/C        T/G        C/C        1.08
#> 25      25 C/C        G/G        T/T       -0.78
```

```
#> 26       26 C/C        G/G        T/C        -0.320
#> 27       27 C/C        G/G        C/C         0.140
```

And below a graphical visualisation of the risk scores from lowest (top) to highest (bottom):

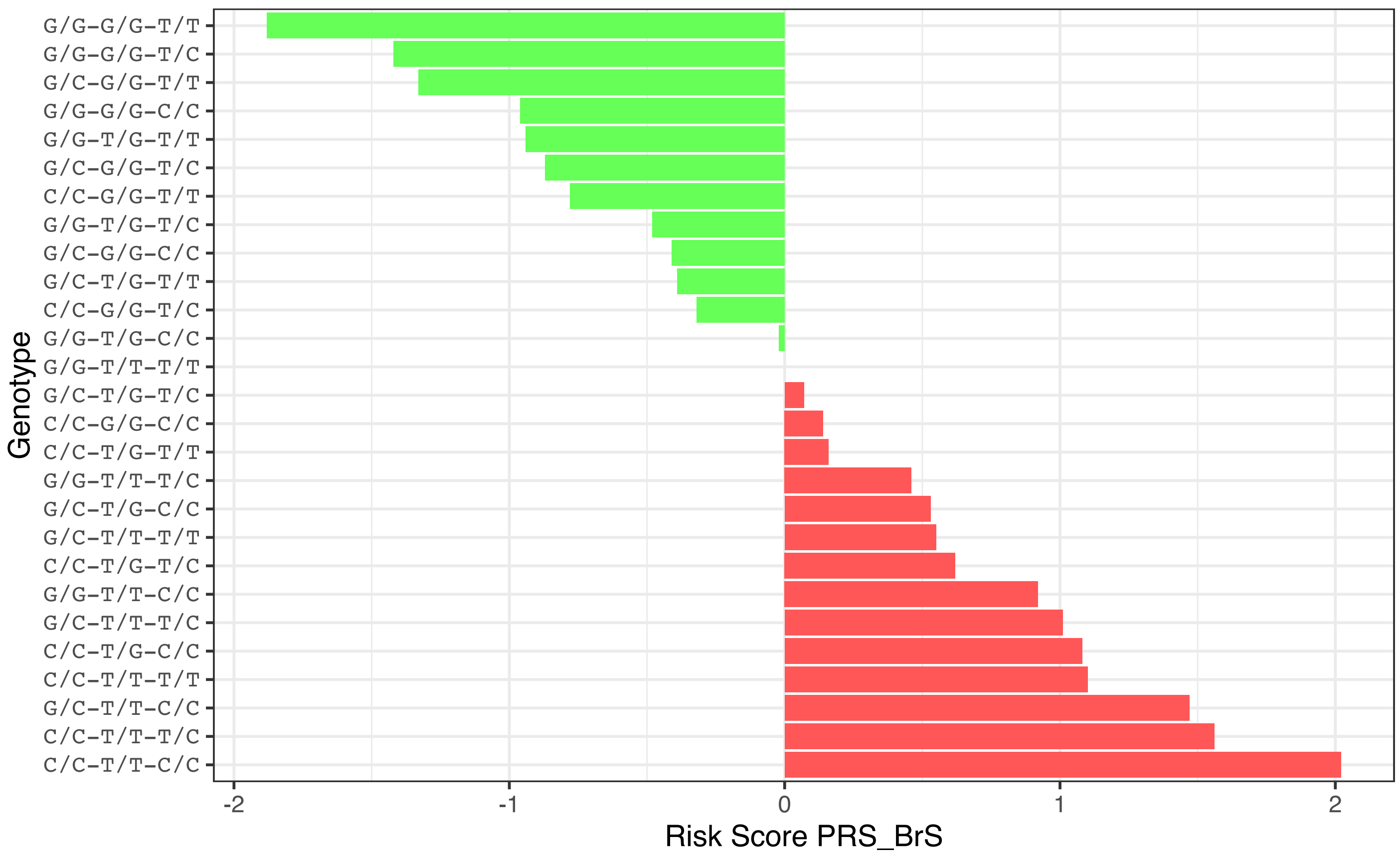

The polygenic risk score spans a range from (almost) -2 to (litle more than) 2. Those genotypes with a negative score carry mostly "protective" alleles, e.g., the most extreme case being `G/G-G/G-T/T` (containing only "protective" alleles)—individuals carrying this genotype have the lowest risk of developing the Brugada syndrome; on the other hand, individuals carrying the genotype `C/C-T/T-C/C` have the highest risk, and should probably be subject to closer clinical follow-up.

Final remarks

The question of how to use the risk scores obtained, namely, how to proceed clinically, is an open and on-going discussion in the medical community that typically needs to be considered in the larger context of each individual characteristics. A good place to start looking into this discussion is the original paper which published this polygenic risk score:
https://academic.oup.com/eurheartj/article/40/37/3097/5559542
(https://academic.oup.com/eurheartj/article/40/37/3097/5559542).

Furthermore, the purpose of this vignette is to show how to start from a trait or disease of interest, and end up by actually calculating the risk scores for particular individuals (if the user has real patient data). In this example, the individuals are hypothetical, and are represented by their in silico constructed genotypes. While trying to apply the same workflow to other traits or diseases, things are likely to be different from this simple example, namely, the number of variants will likely be much larger, and the polygenic risk score model applied could be more complex than the simple additive architecture assumed here. Also, you will probably want to take advantage of a dedicated tool that specialises in score computation instead of using your own custom R code, e.g., you could use the popular bigsnpr (https://privefl.github.io/bigsnpr/articles/LDpred2.html) package.
